# Randomized controlled trial to compare functional outcomes between Locking compression Plates and Retrograde Intramedullary Nails in distal femur fractures

**DOI:** 10.12669/pjms.42.2.13605

**Published:** 2026-02

**Authors:** Aimal Sattar, Ayesha Imtiaz, Syed Imran Bukhari

**Affiliations:** 1 Aimal Sattar, FCPS. Department of Orthopedics, Lady Reading Hospital, Peshawar, Pakistan; 2 Ayesha Imtiaz, PhD. Khyber Medical University, Institute of Public Health, Social Sciences, IPH&SS, Peshawar, Pakistan; 3 Syed Imran Bukhari, FCPS. Department of Orthopedics, Lady Reading Hospital, Peshawar, Pakistan

**Keywords:** Distal femur fracture, Retrograde intramedullary nail, Locking compression plate

## Abstract

**Objective:**

To evaluate the functional result at six months for adult patients with 33A2/3 distal femur fractures treated with locking compression plates (LCP) versus retrograde intramedullary nails (RIMN).

**Methodology:**

A Randomized Controlled Trial was conducted in Department of Trauma & Orthopedics, Lady Reading Hospital Peshawar, from February 2022 to 2024. A total number of 96 patients, aged ≥ 18 years with close AO 33A2/3 distal femur fractures were randomized. Pathological fractures, periprosthetic fractures, patients with metabolic bone or prior knee arthritis or vascular damage requiring repair or underlying deformity in the affected limb precluding use of either implant or drug abuse or unfitness for anesthesia were excluded. The functional outcome was measured through Wilde modification of Neer knee score using Mann-Whitney U test for comparisons. RUST score was used to assess fracture healing. HRQoL was assessed using PROMIS-10.

**Results:**

Out of 96 patients, 19 were female and 77 males. Mean age of the patients was 38.68±15.45 (18-78). Mann-Whitney U test was used for treatment comparison using Wilde modification of Neer knee score and the p value was 0.968. At 6 months, the healing rate was 82.5% in RIMN group and 71.79% in LCP group, according to RUST criteria. Mann-Whiney U test was used to compare physical and mental HRQoL scores between the groups and the p value was 0.028 and 0.122 respectively.

**Conclusion:**

There was no significant difference in functional outcome using Wilde modification of Neer knee score.

**Registration No.:** RCT Trial (ACTRN12622000229774).

## INTRODUCTION

Distal femur fractures are common injuries. Of all fractures, distal femur fractures comprise 0.4% and 3% of femur fractures.^1^,^2^ High energy fractures occur in the younger population, while elderly patients suffer low energy fractures. Management of these fractures is still evolving. Two different concepts have been utilized to treat these fractures; extra-medullary and intra-medullary implants. The main advantages of intramedullary implants over extramedullary implants are that they can be inserted through 2-3 cm incisions versus 10-15 cm for extramedullary implants, and they are load-bearing because they are placed along the anatomical axis, so bending forces are reduced with nails versus plates. Nailing distal femur fractures, on the other hand, requires accessing the knee joint, which might lead to infection or articular surface injury.^3^

Nailing is a more technically challenging process that necessitates close attention to the entrance point, pathway, and reduction of fracture throughout the procedure. Locking compression plates are extra-medullary implants which can be used via open or minimally invasive techniques, they are technically less demanding than nails but require more dissection than nails. Literature regarding claimed superiority of nail over plate or vice versa is equivocal.^4^ One meta-analysis found that the available data is insufficient to guide current clinical practice.^5^ The meta-analysis included two trials that looked at functional outcomes: one compared surgical and non-surgical treatment outcomes, and the other assessed retrograde nail vs fixed angle plate outcomes.

**Fig.1:**
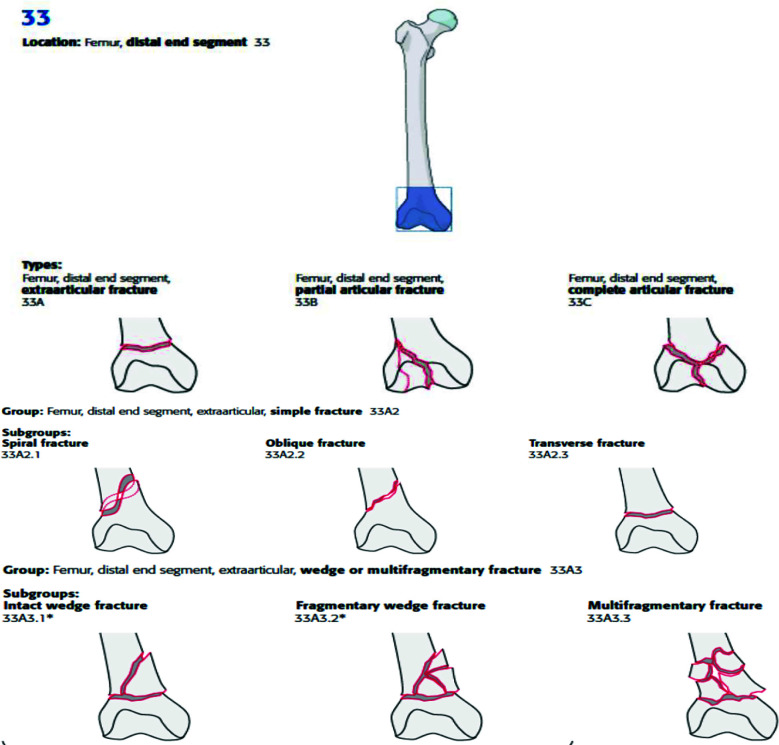
https://surgeryreference.aofoundation.org/orthopedic-trauma/adult-trauma/distal-femur

Distal femur fractures are classified as AO Type 33A2/3 (Fig.1).^6^ Griffin et al. reported the feasibility of conducting an RCT on nail versus plate in distal femur fractures. Their findings demonstrated that their trial design is not likely to be successful on a large scale and concluded that the biggest impediments to recruitment were differences in fracture management between centers, surgeons unsure about using both interventions and lack of research culture.^7^ They included all distal femur fractures irrespective of whether they were intra-articular or extra-articular. Surgeons may have lacked equipoise in managing complex intra-articular fractures, for which less demanding procedures are available. As per Helfet et al, retrograde nails should not be used for type B distal femur fractures, type C3 comminuted fractures, or low transcondylar fractures, because of the risk of articular surface injury and implant instability.^8^ Recently, Dunbar et al published their study comparing results of LCP vs RIMN in AO type A, C1 and C2 with or without total knee prosthesis, showing no difference in functional scores between the groups.^9^ Since ours is a single center study, performed in one of the largest academic teaching hospitals in Pakistan, the lack of cohesion was minimized. By limiting our population to extra-articular fractures, we thought there will be a better equipoise between the two treatments.

### Hypothesis:

The functional outcome at six months using RIMN is different than LCP in the treatment of extra-articular distal femur fractures, based on Wilde’s modification of the Neer knee score.

## METHODOLOGY

A two-arm parallel, Randomized Controlled Trial (ACTRN12622000229774) was conducted at Department of Trauma & Orthopedics, Lady Reading Hospital, Peshawar, an academic teaching hospital for 12 months (February 2022 to 2024).

### The inclusion criteria:

All patients ≥ 18 years old with AO 33A2/3 distal femur fractures with ASA grade 1-3, presenting within seven days of injury.

### The exclusion Criteria:

Unfitness for anaesthesia, pathological fractures, periprosthetic fractures, open fractures, prior knee arthritis, fractures needing correction of vascular damage, an underlying abnormality in the affected limb that would prevent IM nailing or plating, or leftover hardware and a metabolic bone disease diagnosis. Patients who were mentally incompetent or addicted to drugs were also excluded.

Patients were followed up for a period of six months. Primary outcome was assessed using Wilde modification of Neer knee score^10^ (Appendix Table-A). Secondary outcomes assessed were adverse events (Appendix Table-B), fracture union using RUST score (Appendix Table-C) and Health related quality of life using PROMIS-10 Global Health Score (Appendix Table-D).

**Table-I:** Basic demographic characteristics.

Mean Age	38.68±15.45 (18-78); All groups
	40±15.84 (RIMN group)
	37.35±15.09 (LCP group)
***Gender***	
Female	19 (19.8%); 12 RIMN: 7 LCP
Male	77 (80.2%); 36 RIMN, 41 LCP
***Fracture characteristics***	
33A2	51; 27 RIMN, 24 LCP
33A3	45; 21 RIMN, 24 LCP
***Co-morbidities***	
None	77
HTN	8
Diabetes	3
Diabetes +HTN	4
Diabetes +HTN+IHD	2
Mean time to surgery	4.05 ± 1.538 days

**Table-II:** Rust score vs Treatment Crosstabulation.

		Treatment		Total
		RIMN	LCP	
RUST score	6	1	2	3
	7	2	1	3
	8	3	5	8
	9	1	3	4
	10	9	8	17
	11	8	15	23
	12	16	5	21
Total		40	39	79

**Appendix Table-A:** Wilde modification of Neer knee score.

Domain	Score	Definition
Pain	4	No pain in all ranges of motion
	3	Pain with normal daily activity
	2	Minimal activity gives pain
	1	Pain at rest
Movement	4	Flexion > 100º; no fixed flexion deformity
	3	Fixed flexion deformity or flexion limited to 70-100º
	2	Flexion to 30-70º
	1	Flexion < 30º
Function	4	Full weight bearing, normal gait, no impairment of function
	3	Limp, no restriction of activity
	2	Requires walking aid
	1	Cannot walk because of fracture
Shortening	4	0 – 0.5 cm
	3	0.5 – 2.5 cm
	2	2.5– 5 cm
	1	>5 cm
Angulation	4	None
	3	<10º
	2	10-15º
	1	>15º

**Appendix Table-B:** Adverse events.

Type of event	Definition
***Intraoperative***	
Nerve injury	Injury to peroneal nerve
Vascular injury	Damage to a major blood vessel during the surgical procedure
Iatrogenic fracture/fracture extension	New fracture or extension of the existing fracture during application of either LCP or RIMN
Fat embolization	Bone marrow entering the blood stream due to RIMN leading to breathing complications
Articular cartilage damage	Damage to distal femur articular cartilage during exposure
Drop in oxygen saturation	Decreased in blood oxygen levels after reaming
Malrotation/malreduction	Coronal or sagittal plane deformity > 10º
Intra articular screw penetration	Screw entering the articular surface
***Postoperative***	
***Infection***	
Superficial infection	Surgical wound with signs of pain, erythema, swelling, warmth, tenderness to palpation, fever (> 38°C) or (purulent) wound drainage affecting only superficial layers
Deep infection	Presence of discharging sinus with evidence of communication to the joint needing reoperation OR use of antibiotics beyond 5 days after surgery
***Hardware failure***	
Construct pullout	Implant no more holding onto the bone
Implant breakage	Breakage of parts of the fixation material
Implant loosening	Failure of the bond between implant and bone, visible on serial radiological examination
Non-union	<10 RUST score on radiographs 6 months after the surgery
Deep venous thrombosis	Blood clot in the leg
Pulmonary embolism	Blood clot in the leg travelling to the lungs with breathing complications
Surgical revision	Need for reoperation from any other cause not specified above such as hematoma, bleeding, hardware removal due to pain, nail impingement at knee etc
Blood loss	Blood loss leading to anemia (Hb<8g/dl) or requiring transfusion

**Appendix Table-C:** RUST score.

Score per cortex	Callus	Fracture line
1	Absent	Visible
2	Present	Visible
3	Present	Invisible with bridging callus Visible with bridging callus

Radiological union: defined as RUST score ≥10.

Radiological union: defined as RUST score ≥10

**Figure:**
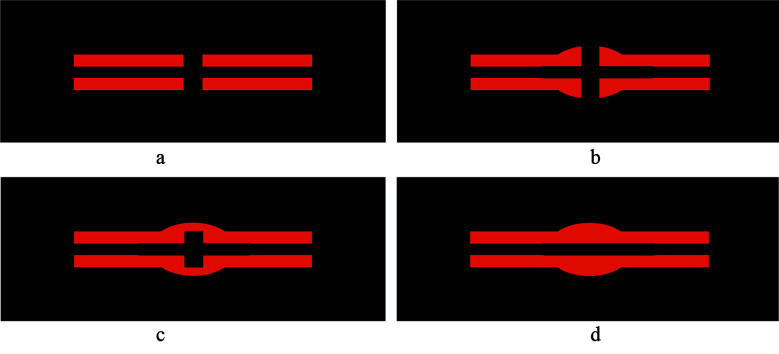


Fracture with fracture line visible and no callus - RUST score 1

Fracture with callus formation and a fracture line – RUST score 2

Fracture with bridging callus but fracture line visible – RUST score 3

Complete bridging of callus with no evidence of fracture line – RUST score 3

**Appendix Table-D:** PROMIS-10 Global Health Score.

		Excellent		Very good		Good			Fair		Poor	
Global 01	In general, would you say your health is ….	ʘ 5		ʘ 4		ʘ 3			ʘ 2		ʘ 1	
Global 02	In general, would you say your quality of life is ……	ʘ 5		ʘ 4		ʘ 3			ʘ 2		ʘ 1	
Global 03	In general, how would you rate your physical health? …..	ʘ 5		ʘ 4		ʘ 3			ʘ 2		ʘ 1	
Global 04	In general, how would you rate your mental health, including your mood and your ability to think? …….	ʘ 5		ʘ 4		ʘ 3			ʘ 2		ʘ 1	
Global 05	In general, how you rate your satisfaction with your social activities and relationships?..	ʘ 5		ʘ 4		ʘ 3			ʘ 2		ʘ 1	
Global 09	In general, please rate how well you carry out your usual social activities and roles. (This includes activities at home, at work and in your community, and responsibilities as a parent, child, spouse, employee, friend etc) ….	ʘ 5		ʘ 4		ʘ 3			ʘ 2		ʘ 1	
		Completely		Mostly		Moderately			A little		Not at all	
Global 06	To what extent are you able to carry out your everyday physical activities such as walking, climbing stairs, carrying groceries, or moving a chair?…	ʘ 5		ʘ 4		ʘ 3			ʘ 2		ʘ 1	
***In the past 7 days ….***												
		Never		Rarely		Sometimes			Often		Always	
Global 10	How often have you been bothered by emotional problems such as feeling anxious, depressed or irritable? ….	ʘ 1		ʘ 2		ʘ 3			ʘ 4		ʘ 5	
		None		Mild		Moderate			Severe		Very severe	
Global 08	How would you rate your fatigue on average? …	ʘ 1		ʘ 2		ʘ 3			ʘ 4		ʘ 5	
Global 07	How would you rate your pain on average? …	ʘ 0	ʘ 1	ʘ 2	ʘ 3	ʘ 4	ʘ 5	ʘ 6	ʘ 7	ʘ 8	ʘ 9	ʘ 10
		No pain										Worst imagi-nablePain

Whelan et al developed the RUST score to determine the healing of tibial fractures after intramedullary nail.^11^ This outcome measure has since been used in multiple studies.^12^,^13^ It has been used in bones other than tibia as well and has been used in both operatively and non-operatively treated fractures.^14^,^15^ RUST score ≥ 10 was considered as radiologically healed fracture for this study.^15^ Neer described a disease-specific measurement outcome for distal femur fracture in 1967 and Wilde modified it in 1989.^16^,^17^ Based on a previous report, the expected average score in the nailing group is 15 points in Wilde’s modification of the Neer score. As there are no studies available on the minimum clinically important difference for Wilde’s modification of the Neer score, we assumed that a difference of two points in the total score will be clinically relevant, which translates to 2-point difference on any subscale or 1-point difference on any two scales. Based on this assumption, the average score in the plating group was expected to be 13 points. We assumed a SD of three points in each group, a non-normal distribution, a nail versus plate ratio of 1:1, a two-sided test, alpha = 5%, power = 80%, and a dropout rate of 15%. The total sample size was calculated at 96 randomized patients (48 per group).

The patient-reported outcome was assessed using the PROMIS-10 Global Health score.^18^ A ten-item survey called the PROMIS-10 evaluates a person’s self-reported physical and mental health as well as their quality of life. This measure uses a T-score metric, where the standard deviation (SD) is represented by 10 and the mean (50) of a pertinent reference population is represented.

**Fig.2:**
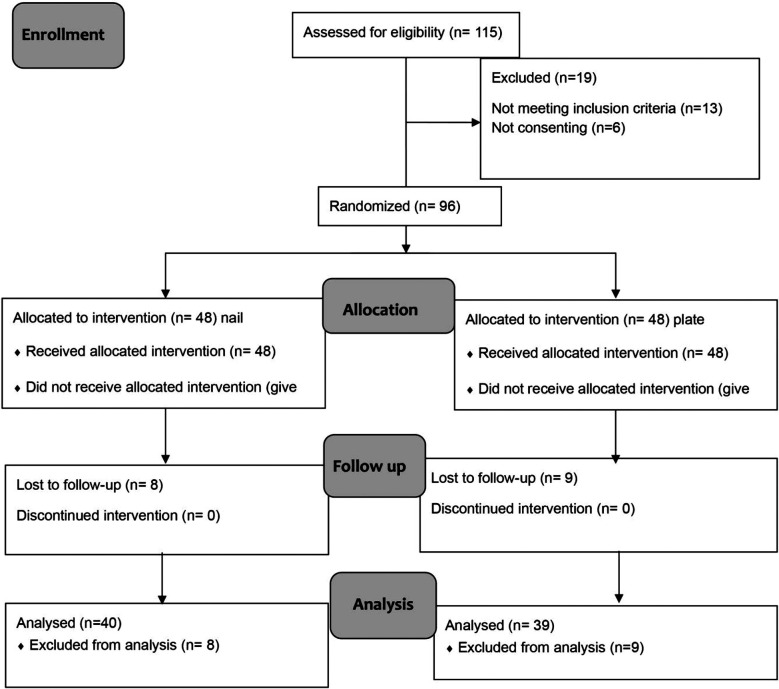
CONSORT Flowchart.

Random assignment using simple random-probability sampling technique was used to place each eligible patient in the LCP or RIMN groups. An independent researcher created the randomization list utilizing random permuted block sizes of six using sealedenvelope.com. The allocation of treatment was requested over the phone from the independent researcher once the patient was in the operating room and under anesthesia for the procedure. A total number of 96 patients were randomized (i.e. enrolled) in a 1:1 ratio between both arms. As soon as a patient was randomized, he/she was followed up along the whole period to allow analysis. (Fig.2, CONSORT Flowchart) Two surgeons with experience in trauma were involved in this study.

A single radiologist was involved in grading of radiographs for RUST score. Because of the variations in implants and wound patterns, neither the patient nor the surgeon could be blindfolded. Patients were kept non-weight bearing for 4-6 weeks and weight bearing was started once evidence of callus was noted on x-rays. Range of motion exercises were started soon after surgery except when bracing was applied occasionally for soft tissues condition. Consent was obtained from every patient before any study-specific procedures or assessments took place.

### Ethical Approval:

The study was approved by LRH IRB, Ref no. 246/LRH/MTI.

## RESULTS

The basic demographic data is shown in Table-I. Kolmogorov-Smirnov test was used to check for normality of the data and the p value was <0.001. Mann-Whitney U test was used for treatment comparison using Wilde modification of Neer knee score and the p value was 0.968 (mean score 15.39±2.875; 15.48 RIMN±2.562: 15.31±3.197 LCP). There were 15 adverse events in LCP group (four non-union, one infection, one fat embolism, three malunion, one intra-articular screw, two construct pull-out, two broken implants and one delayed union) while nine adverse events in RIMN group (2 non-union, two infection, one fat embolism, two malunion, one construct pull-out and one broken implant). At six months, 82.5% fractures had healed in RIMN group, while 71.79% fractures healed in LCP group, according to RUST criteria. (Table-II) Mann-Whiney U test was used to compare physical and mental HRQoL scores between the groups and the p value was 0.028 and 0.122 respectively. The mean PROMIS mental score in RIMN group was 47.147±8.29, while in LCP group, it was 44.259±9.17. The mean PROMIS physical score in RIMN group was 49.250±8.00, while in LCP group, it was 45.772±7.94. There was no significant difference (p=0.383) when comparing outcome scores across the different fracture groups.

## DISCUSSION

Distal femur fractures can be managed with either extramedullary or intramedullary implants. Our trial has shown that there was insignificant difference in functional outcome using either LCP or RIMN in managing these fractures. Mean age of the patients in this study was 38.68±15.45 (18-78). Griffin et al reported mean age of 70.1 in RIMN group against 78.7 in LCP group in their feasibility RCT. The mean age in the study by Dunbar et al 9 was 51 years (range 16-90 years). Our study population is younger in comparison to the previously reported studies. Of the 96 patients, 19 (19.8%) were female while 77 (80.2%) were male. In the study by Dunbar et al,^9^ there were 71 men and 55 women (of the 126 completing follow-up) evenly distributed across the groups. Griffin et al reported 16 female and seven male participants. Due to peculiar socio-economic culture of our population, male participants outnumber female participants in the current study.^7^

According to AO classification, there were 51 patients in 33A2 group and 45 patients in 33A3 group in our study. In the study by Griffin et al, the fractures included were AO/OTA type A1 (12 fractures), A2 (five fractures), A3 (one fracture), B1 (one fracture), C2 (two fractures), C3 (3 fractures). Dunbar et al included fracture of the metaphyseal distal femur with or without simple intra-articular extension (OTA/AO 33-A, 33-C1 or 33-C2) and with or without total knee arthroplasty.

Mann-Whitney U test was used in the present study for treatment comparison using Wilde modification of Neer knee score and the p value was 0.968. Dunbar et al reported Short Musculoskeletal functional assessment (SMFA) score of 22.2 for nailing and 26.8 for plating group (p=0.29), Bother index (22.9 nail vs 28.5 plate; p=0.3), EQ Health (79.1 nail vs 72 plate; p=0.11), EQ Index (0.76 nail vs 0.7 plate; p=0.25). Griffin et al reported EQ-5D-5L values of 0.38 for nailing group and 0.37 for plating group. Our results are in agreement with previously published studies comparing these two treatment options in that the difference in primary outcome was insignificant. There were 15 adverse events in LCP group while 9 adverse events in RIMN group. Dunbar et al reported an adverse event rate of 20%, which was not statistically different between the groups. Griffin et al reported three complications in nailing group, namely pneumonia, urinary tract infection and myocardial infarction while two patients each in both the groups needed blood transfusion.^7^,^9^

At six months, 82.5% fractures were healed in RIMN group, while 71.79% fractures healed in LCP group, according to RUST criteria. RIMN results in indirect healing, while LCP application may result in indirect or direct healing. Radiological assessment may overestimate non-healing in direct healing despite clinical union. At the protocol stage, we chose different timelines to see which modality of treatment leads to faster rate of union, but unfortunately, we could not implement the protocol fully on account of non-adherence due to poor follow up and thus we were unable to know the difference and are reporting results at six months only (ACTRN12622000229774). Mann-Whiney U test was used to compare physical and mental HRQoL scores between the groups and the p value was 0.028 and 0.122 respectively. To date, this is the first study which has used PROMIS tool for HRQoL assessments in distal femur fractures.

### Strengths and limitations:

Strengths include randomized trial to assess the outcome in extra-articular distal femur fractures and the use of patient reported outcome measures. Limitations include single center study with a relatively short follow-up period and non-adherence to the RCT protocol in its entirety and more importantly, 17.7% loss to follow-up to accurately assess the primary outcome.

## CONCLUSIONS

There was insignificant difference in functional outcome after use of either LCP or RIMN using the Wilde modification of Neer knee. Further studies with more robust clinical outcome instruments and longer follow up are recommended to inform the decision making in the management of extra-articular distal femur fractures.
